# Circulating Gal‐3 and sST2 are associated with acute exercise‐induced sustained endothelial activation: Possible relevance for fibrosis development?

**DOI:** 10.1113/EP091277

**Published:** 2023-08-12

**Authors:** Julia M. Kröpfl, Fernando G. Beltrami, Hans‐Jürgen Gruber, Arno Schmidt‐Trucksäss, Thomas Dieterle, Christina M. Spengler

**Affiliations:** ^1^ Division of Sport and Exercise Medicine, Department of Sport, Exercise and Health University of Basel Basel Switzerland; ^2^ Exercise Physiology Lab, Institute of Human Movement Sciences and Sport ETH Zurich Zurich Switzerland; ^3^ Clinical Institute of Medical and Chemical Laboratory Diagnostics Medical University of Graz Graz Austria; ^4^ Foeldiklinik GmbH&Co KG Hinterzarten Germany; ^5^ Department of Clinical Research University Hospital Basel Basel Switzerland; ^6^ Zurich Center for Integrative Human Physiology (ZIHP) University of Zurich Zurich Switzerland

**Keywords:** early diastolic filling ratio, endothelial cells, galectin‐3, high‐intensity interval exercise, myocardial contractility index, soluble suppression of tumorigenicity 2

## Abstract

Long‐term, intense endurance exercise training can occasionally induce endothelial micro‐damage and cardiac fibrosis. The underlying mechanisms are incompletely understood. Twenty healthy, well‐trained male participants (10 runners and 10 cyclists) performed a strenuous high‐intensity interval training (HIIT) session matched by age, height, weight and maximal oxygen consumption. We assessed the acute exercise response of novel cardiac biomarkers of fibrosis [e.g., galectin‐3 (Gal‐3) and soluble suppression of tumorigenicity 2 (sST2)] per exercise modality and their relationship with haemodynamic contributors, such as preload, afterload and cardiac contractility index (CTi), in addition to endothelial damage by sustained activation and shedding of endothelial cells (ECs). Serum Gal‐3 and sST2 concentrations were investigated by enzyme‐linked immunosorbent assays; haemodynamics were analysed via impedance plethysmography and circulating ECs by flow cytometry. The Gal‐3 and sST2 concentrations and ECs were elevated after exercise (*P* < 0.001), without interaction between exercise modalities. Circulating Gal‐3 and sST2 concentrations both showed a positive relationship with ECs (*r*_rm_ = 0.68, *P* = 0.001 and *r*_rm_ = 0.57, *P* = 0.010, respectively, both *n* = 18). The EC association with Gal‐3 was significant only in cyclists, but equally strong for both modalities. Gal‐3 was also related to exercise‐induced CTi (*r*_rm_ = 0.57, *P* = 0.011, *n* = 18). Cardiac wall stress is increased after an acute HIIT session but does not differ between exercise modalities. Exercise‐released Gal‐3 from cardiac macrophages could very probably drive systemic endothelial damage, based on an enhanced CTi. The importance of acute exercise‐induced vascular resistances and cardiac contractility for the release of fibrotic biomarkers and any long‐term pathological endothelial adaptation should be investigated further, also relative to the exercise modality.

## Abstract (highlights)

- **What is the central question of this study?**
  Circulating biomarkers of cardiac wall stress and fibrosis are influenced by physical exercise. The underlying mechanisms per exercise modality are still unclear.
- **What is the main finding and its importance?**
  We show that galectin‐3 (Gal‐3) and soluble suppression of tumorigenicity 2 (sST2) are increased after acute exercise but do not differ between running and cycling. One haemodynamic contributor to the secretion of Gal‐3 is an enhanced cardiac contractility. Acute exercise‐released Gal‐3 and sST2 are linked to sustained endothelial activation and cell shedding. This could be relevant in the context of fibrosis development and could identify athletes at risk for pathological endothelial adaptations.

## 1 INTRODUCTION

Regular physical exercise is generally recognized to have beneficial effects on cardiovascular health. However, there might be a limit to benefit in the exercise dose–response relationship. Excessive, intense endurance exercise training can induce endothelial micro‐damage, occasionally leading to pathological cardiac remodelling (Parry‐Williams & Sharma, 2020) and a higher incidence of left ventricular myocardial fibrosis (Maceira et al., 2023), but the clinical relevance of these results is controversial (Maceira et al., 2023). Information on the effects of single exercise training sessions might help to decipher the possible acute impacts on the heart, which could, in the long term, lead to a sustained stimulation of endothelial cells (ECs) and early fibrotic endothelial‐to‐mesenchymal transition (Hsu et al., 2019).

So far, endurance exercise lasting >30 min has been investigated using common biomarkers, such as cardiac troponins (Donaldson et al., 2019) or N‐terminal prohormone of brain natriuretic peptide (Urhausen et al., 2004). Novel biomarkers of cardiac wall stress and fibrosis, such as galectin‐3 (Gal‐3) (Hrynchyshyn et al., 2013; Sygitowicz et al., 2021) and soluble suppression of tumorigenicity 2 (sST2) (Biaggi et al., 2019), have emerged and might have the potential to add important information to characterize cardiac strain after acute exercise, also in different endurance exercise modalities (Le Goff, Farre Segura, et al., 2020).

Cardiac macrophages are the main sources of circulating Gal‐3, which are activated by inflammatory processes induced by ventricular overload. The main action of Gal‐3 is to bind to and activate fibroblasts that form collagen and scar tissue, leading to progressive cardiac fibrosis (Hrynchyshyn et al., 2013). Expression and secretion of sST2 in and from cardiac cells has been described in the response to myocardial stress and biomechanical overload (Dudek et al., 2020). Elevated concentrations of Gal‐3 and sST2 were demonstrated after acute exercise over a wide range of durations and intensities, such as 1 h [75% maximal oxygen consumption (${\dot{V}}_{O_{2}\max}$)] runs (Le Goff, Kaux, et al., 2020), 4 h runs (Kaleta‐Duss et al., 2020; Le Goff, Kaux, et al., 2020), half‐marathon (Vassalle et al., 2018), marathon (Aengevaeren et al., 2019) and 8 h ultra trail runs (Le Goff, Kaux, et al., 2020) in a middle aged population when compared with established reference values (Agnello et al., 2017; Biaggi et al., 2019).

These novel circulating biomarkers of cardiac wall stress and fibrosis have not been investigated after acute high‐intensity cycling so far. Based on echocardiographic results, cycling might even induce higher postexercise sST2 and Gal‐3 concentrations than running, because left ventricular wall thickness can be increased disproportionately in experienced cyclists owing to both volume and pressure overload as a result of a combination of mainly isotonic exercise with isometric work of the upper part of the body when compared with runners (Fagard et al., 1984). Potential concentration differences between running and cycling could also be explained by different haemodynamic consequences (Le Goff, Farre Segura, et al., 2020), because isotonic work is associated with a substantial increase in cardiac output (CO) and reduction in peripheral vascular resistance, whereas isometric work is characterized by a smaller increase in CO and only a transient increase in peripheral resistance. This would subsequently influence blood flow, whereby low oscillatory flow, with changes in direction and magnitude, is known to induce a pro‐inflammatory, profibrotic vessel state (Kruger‐Genge et al., 2019). However, single contributors to Gal‐3 and sST2 secretion, such as systemic vascular resistances (preload and afterload) or myocardial contractility measured by impedance cardiography, are yet to be elucidated.

Moreover, the number of ECs shed from the vasculature by acute exercise as marker of endothelial damage could help in prediction of early fibrosis (Hsu et al., 2019), especially in combination with Gal‐3 (Berezin et al., 2019). Importantly, a recent study (Vertes et al., 2022) found that Gal‑3 levels, but not sST2 levels, exhibited a significant positive association with echocardiographic markers of myocardial mechanics, such as left ventricular global longitudinal strain. The usefulness of Gal‐3 for the screening and early diagnosis of cardiac fibrosis was also hypothesized by the authors.

The aim of this study was to compare Gal‐3 and sST2 as novel serum biomarkers for cardiac remodelling and fibrosis at baseline and after acute high‐intensity interval training (HIIT) exercise in well‐trained endurance athletes, also relative to the exercise modality. Runners and cyclists were matched for age, height, weight and peak oxygen consumption (${\dot{V}}_{O_{2}\mathrm{peak}}$). A further aim of the study was to determine the associations between the early diastolic filling ratio (EDFR; a surrogate for preload), systemic vascular resistance index (SVRi; a surrogate for afterload) and the myocardial contractility index (CTi; independent of pre‐ or afterload), with circulating serum cardiac biomarkers and EC shedding measured by flow cytometry.

We hypothesized that acute exercise would elevate both sST2 and Gal‐3 and to a greater extent in cyclists compared with runners. Biomarker concentrations would generally be associated with EDFR, SVRi, CTi and ECs, but to a greater extent in cyclists owing to the specific cardiac load conditions found in these athletes.

## 2 MATERIALS AND METHODS

### 2.1 Ethical approval

Informed consent was obtained from each participant in writing, and the study protocol conformed to the ethical guidelines of the 1975 *Declaration of Helsinki* as reflected in a priori approval by the cantonal Ethics Committee Zurich, including blood sampling and further use of the samples collected for research purposes (BASEC 2017‐00128). Please see for details: https://clinicaltrials.gov/study/NCT03155152.

### 2.2 Study design

This is a post hoc analysis of a larger project described elsewhere (Beltrami et al., 2021; Kropfl et al., 2020, 2021). In brief, healthy, well‐trained runners and cyclists were recruited to measure the acute cardiorespiratory responses to different sessions of HIIT. They did not show any obvious sign of cardiovascular disease. To be included, participants needed to have an average training volume of ≥150 km/week for cyclists and 40 km/week for runners in at least three weekly training sessions in the 3 months before the study (Beltrami et al., 2021). For the current post hoc investigation, serum samples from 20 male participants were analysed and the results related to unpublished data on EDFR, myocardial CTi, SVRi and indicators of sustained endothelial activation (i.e., the number of circulating ECs). Samples from runners (*n* = 10) and cyclists (*n* = 10) were matched for participants’ age, height, weight and ${\dot{V}}_{O_{2}\max}$.

All sessions took place at the same time of day (±1 h), and participants had to refrain from intensive sports for 48 h and to avoid sports for 24 h before each visit. In addition, they were asked to sleep for ≥7 h on the 2 nights preceding the visits and to avoid taking caffeinated products on the testing day or eating for 2 h before a test.

### 2.3 Exercise trial

All exercise sessions took place in standardized laboratory conditions in a temperature‐controlled room. Participants underwent an acute exercise session of traditional HIIT. Acute exercise consisted of four bouts of 4 min high‐intensity cycling/running interspersed with 3 min of low‐intensity exercise. The first bout was preceded by a 5 min warm‐up, with 3 min at 100 W/8 km/h and 2 min at 50% of the difference between the first stage and the first bout, and followed by 3 min of cool‐down. The average speed or power of intense phases was 85% of maximal speed or power from a previous incremental test, depending on the exercise modality. The incremental test had the following profile for cycling and running, respectively: 100 W + 20 W/min or 10 km/h + 1 km/h/min. Ventilation and gas exchange (e.g., oxygen consumption) were measured breath by breath by a metabolic cart (Oxycon Pro; Jaeger). Data were averaged over 5 s intervals for subsequent analyses. Peak values in the incremental test (e.g., ${\dot{V}}_{O_{2}\max}$) were determined as the highest 30 s moving average (5 s intervals) during the test.

Given that only the 16 min at high intensity were expected to have an influence on the outcome variables (Adams, 2018), the 17 min of low‐intensity exercise were not characterized.

### 2.4 Serum and EC isolation and characterization

Before the warm‐up and 10 min after the last high‐intensive exercise bout, 23 mL of blood was withdrawn by venipuncture. Serum was isolated (1500*g*, 10 min, 23°C) and kept frozen at −80°C until analysis. Three hundred microlitres of whole blood was kept for haematological analysis (ADVIA 2120i; Siemens, Zurich, Switzerland), and the remaining volume was used for a standard Ficoll gradient centrifugation (Histopaque; Sigma‐Aldrich, Switzerland; catalogue no. 10771) within 2 h after blood withdrawal to isolate peripheral blood mononuclear cells (MNCs) for EC analysis by flow cytometry in all subjects.

### 2.5 Flow cytometry

**FIGURE 1:**
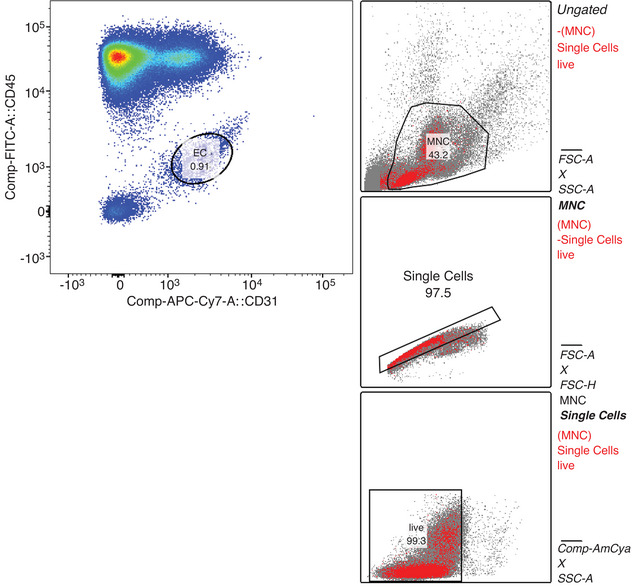
Exemplary flow cytometry gating protocol for the assessment of sustained endothelial activation. After duplet exclusion and extraction of live cells, total mononuclear cells (MNC) were gated for CD31^+^/CD45^−^ endothelial cells (EC). Numbers extracted are given as a percentage of the parent population. CD, cluster of differentiation; FSC‐A, forward scatter ‐area; FSC‐H, forward scatter ‐ height; SSC‐A, side scatter ‐ area, Comp, compensated; FITC, fluorescein isothiocyanate; APC‐Cy7, allophycocyanin cy7 tandem conjugate; AmCya, anemonia majano cyan fluorescent protein.

Sample preparation for flow cytometry is described in detail elsewhere (Kropfl et al., 2020). The specific gating strategy to assess mature ECs is depicted in Figure 1. The main acquisition gate was established based on forward and side scatter characteristics, including lymphocytes and MNCs but excluding debris. At least 200 000 MNCs were acquired. After doublet exclusion and gating for live cells, ECs were detected as CD31^+^/CD45^−^ cells, as a percentage of live MNCs. Final flow cytometry data were investigated with a separate analysis tool (FlowJo, Oregon). Estimates of the number of ECs were presented as cells per microlitre, calculated by multiplying respective total EC proportions by the absolute number of MNCs in peripheral blood measured by a standard haematology analyser (Kropfl et al., 2020, 2019).

### 2.6 Determination of Gal‐3 and sST2

Both Gal‐3 and sST2 were analysed in a blinded manner using commercial enzyme‐linked immunosorbent assays from R&D Systems (Abingdon, UK). Serum samples were prepared as recommended by the manufacturer and analysed on a Chameleon plate reader system (Hidex).

### 2.7 Vascular resistance at baseline

The EDFR, myocardial CTi and SVRi were assessed by impedance plethysmography (Physioflow; Manatec Biomedical, Poissy, France) before the acute exercise, and only CTi also during the bouts of HIIT. After skin preparation and electrode placement according to the manufacturer's instructions, participants were seated quietly in an armchair. Initially, arterial blood pressure was measured in duplicate on the left arm using a commercially available device (Mio Star CP500, Zurich, Switzerland). Then, automated calibration of the impedance plethysmography system was done over 30 heart cycles. Subsequently, data were acquired beat by beat for the following 3 min, with the final 60 s taken as representative of rest. Data were analysed in 10 s averages.

The EDFR, CTi and SVRi were calculated automatically by the Physioflow software (Gordon et al., 2018). The EDFR was measured on the d*Z*/d*t* and was defined as the ratio of the O wave to the S wave. The SVRi was calculated as 80 × (MAP − CVP)/CI, where MAP is the mean arterial pressure calculated from the systolic and diastolic blood pressure entered by the user, CVP is the central venous pressure, which was, by default, set as 7 mmHg during the calibration procedure, and CI is the cardiac index. The Physioflow device was found to be acceptable for measuring heart rate, stroke volume, CO and CTi during rest and interval exercise, with intraclass correlation coefficients (ICCs) > 0.75 (except ICC_CTi_ = 0.65 at rest), whereas EDFR, SVR and CI showed only moderate reliability at rest (ICC_EDFR_ = 0.38, ICC_SVR_ = 0.53 and ICC_CI_ = 0.60). Careful consideration should be given to the use of variables with ICC < 0.75 (Gordon et al., 2018).

### 2.8 Statistics

Normally distributed data were represented as the mean ± SD and non‐normally distributed data as the median and interquartile range. Data were analysed for the total group and per exercise modality. A priori sample size calculation revealed a necessary *n* = 8 per group in order to detect a postexercise difference between modalities regarding a common cardiac biomarker (N‐terminal prohormone of brain natriuretic peptide) using Student's unpaired *t*‐test, two‐tailed, α = 0.05, 1 − β = 0.8, effect size = 1.52, in ultra‐trail runners (postexercise mean = 325 ng/L, SD = 220 ng/L; Le Goff, Kaux, et al., 2020) and cyclists (postexercise mean = 80.64 ng/L, SD = 60.00 ng/L; Le Goff et al., 2015). Repeated‐measures two‐way ANOVA (repeated effect: exercise; group effect: exercise modality) with Bonferroni post hoc comparisons was used for the analysis of cell and serum parameters. Between‐group comparisons for subject characteristics, EDFR, CTi and SVRi were performed using Student's unpaired *t*‐tests. Pearson, Spearman or repeated‐measures correlation (coefficient *r*_rm_) analyses determined the relationships between variables (Bakdash & Marusich, 2017). A *P*‐value of <0.05 was considered significant.

## 3 RESULTS

### 3.1 Study population

Baseline participant characteristics are given in Table 1. Groups were well balanced for age, body mass index and fitness. Baseline SVRi, Gal‐3, sST2 and EC were comparable between modalities.

**TABLE 1:** Baseline and postexercise results.

Exercise modality	BMI (kg/m^2^)	Age (years)	EDFR (%)	CTi	SVRi (dyn s/cm^5^/m^2^)	Gal‐3 (ng/mL)	sST2 (ng/mL)	ECs (cells/μL)
Running								
Baseline	22.3 ± 1.2	30.6 ± 3.9	48.4 ± 3.7	271.7 ± 45.8	2278.7 ± 421.5	3.8 ± 1.2	17.0 ± 5.5	6.7 ± 2.9
Postexercise				424.9 ± 149.0		5.5 ± 1.7	18.3 ± 6.1	9.0 ± 5.0
Cycling								
Baseline	23.0 ± 1.6	29.0 ± 4.7	53.6 ± 10.0	185.2 ± 52.7	2517.0 ± 497.0	4.4 ± 1.2	16.8 ± 6.0	7.4 ± 4.7
Postexercise				317.4 ± 129.7		5.9 ± 1.5	18.0 ± 5.6	12.4 ± 7.7
Baseline comparison								
*t* (d.f.)	−1.221 (18)	0.825 (18)	−1.535 (16)	3.728 (16)	−1.102 (16)	−1.14 (18)	0.096 (18)	−0.221 (17)
*P*‐value (two‐sided)	0.238	0.420	0.144	**0.002**	0.287	0.269	0.924	0.827
95% Confidence interval	−2.07, 0.55	−2.47, 5.67	−12.41, 2.00	37.34, 135.77	−696.89, 220.29	−1.70, 0.50	−5.16, 5.65	−4.13, 3.34
Both								
Baseline	22.6 ± 1.4	29.8 ± 4.3	50.7 ± 7.4	233.3 ± 64.9	2384.6 ± 458.9	4.1 ± 1.2	16.9 ± 5.6	7.1 ± 3.8
Postexercise				377.2 ± 147.3		5.7 ± 1.6	18.1 ± 5.7	10.7 ± 6.5

*Note*: Values are reported as the mean ± SD; *n*_Running_ = 10, *n*_Cycling_ = 10, except EDFR, CTi and SVRi *n*_Cycling_ = 8 owing to technical issues of the physioflow assessment and EC *n*_Running_ = 9 and EC n_Cycling_ = 9 owing to incomplete antibody staining. Baseline differences between modalities were analysed by Student's unpaired *t*‐tests.

Abbreviations: BMI, body mass index; CTi, contractility index; EDFR, early diastolic filling ratio; Gal‐3, Galectin‐3; sST2, soluble suppression of tumorigenicity 2; SVRi, systemic vascular resistance index.

### 3.2 Exercise trial

The exercise session specific power was 87.9 ± 4.7% of peak power output from a previous incremental test characterized in Table 2. In the HIIT exercise session, runners and cyclists performed 89.9 ± 4.0% and 85.9 ± 4.6% of peak power output, respectively. The HIIT session was comparable between running and cycling regarding percentage peak power output, relative mean oxygen consumption, mean heart rate, breathlessness, respiratory exertion and leg exertion (Table 2).

**TABLE 2:** Characterization of the incremental test and the acute exercise trial.

	HR_max_ (INC) (beats/min)	${\dot{V}}_{O_{2}\max}$ (INC) (mL/min/kg)	HR_mean_ (HIIT) (beats/min)	${\dot{V}}_{O_{2}\mathrm{mean}}$ (HIIT) (mL/min/kg)	Breathlessness (HIIT)	Respiratory exertion (HIIT)	Leg exertion (HIIT)
Running	181.5 ± 7.2	59.0 ± 3.7	170.1 ± 5.6	53.0 ± 3.1	4.4 ± 3.0	4.8 ± 2.2	5.2 ± 2.3
Cycling	188.7 ± 9.8	62.0 ± 3.7	170.4 ± 9.9	53.3 ± 3.9	3.2 ± 3.1	6.1 ± 1.4	6.7 ± 1.2
*t* (d.f.)	−1.691 (14)	−1.821 (18)	−0.086 (18)	−0.172 (18)	0.914 (18)	−1.457 (18)	−1.945 (13.7)
*P*‐value (two‐sided)	0.113	0.085	0.932	0.866	0.373	0.162	0.073
95% Confidence interval	−16.30, 1.93	−6.47, 0.46	−7.86, 7.24	−3.57, 3.03	−1.60, 4.06	−3.00, 0.54	−3.33, 0.17

*Note*: Values are reported as the mean ± SD; *n*_Running_ = 10, *n*_Cycling_ = 10. Differences between modalities were analysed by Student's unpaired *t*‐tests.

Abbreviations: HIIT, high‐intensity interval training session; HR_mean_, mean heart rate; INC, incremental test; ${\dot{V}}_{O_{2}\max}$, maximal oxygen consumption; ${\dot{V}}_{O_{2}\mathrm{mean}}$, mean oxygen consumption.

### 3.3 Cardiovascular biomarkers

**FIGURE 2:**
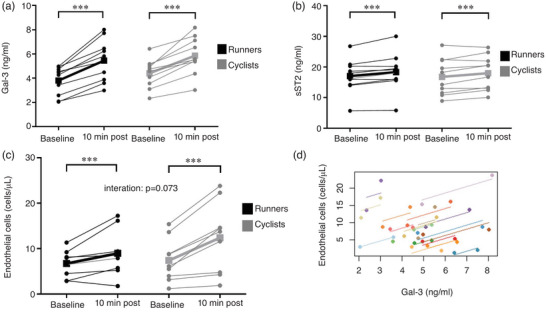
Exercise‐induced novel cardiac biomarkers and sustained endothelial activation. (a, b) Galectin‐3 (Gal‐3; a) and soluble suppression of tumorigenicity 2 (sST2; b) were assessed at baseline and postexercise. Results were significantly increased postexercise, without interaction between groups. Differences in colour indicate the two different exercise modalities, both *n* = 10. (c) Sustained endothelial activation (EC shedding) was also significantly elevated postexercise, with no interaction for exercise modalities, both *n* = 9. (d) Circulating Gal‐3 concentrations were significantly related to sustained endothelial activation by circulating endothelial cells (ECs; *r*_rm_ = 0.68, *P* = 0.001, *n* = 18). Significant differences are indicated by ^***^ *P* < 0.001 and were assessed by mixed ANOVA with one repeated factor (exercise) and one group factor (exercise modality). Squares connected by thick lines indicate mean values of each exercise modality per time point. Parameter association was addressed by repeated‐measures correlation analysis.

There was a significant increase in Gal‐3 from 4.11 ± 1.18 to 5.66 ± 1.59 ng/mL (39.5 ± 17.6%) and in sST2 from 16.92 ± 5.60 to 18.14 ± 5.73 ng/mL (7.9 ± 6.5%) shortly after exercise cessation, with no interaction between exercise modalities (Figure 2a,b; Table 1). For both Gal‐3 and sST2, a significant main effect of exercise was observed (both *P* < 0.001). Acute exercise‐induced changes for Gal‐3 and sST2 were comparable between running and cycling (44.9 ± 17.9% vs. 34.2 ± 16.4%, *P* = 0.179; and 7.2 ± 5.4% vs. 8.6 ± 7.7%, *P* = 0.636, respectively).

### 3.4 Endothelial cell concentrations

Endothelial cell concentrations increased significantly, from 6.9 ± 3.4 to 10.7 ± 6.5 cells/μL (48.8 ± 44.1%; Figure 2c; Table 1). There was a main effect of exercise (*P* = 0.001). No interactions for exercise modalities were found (*P* = 0.073). Acute exercise‐induced changes for ECs were comparable between running and cycling (30.2 ± 40.8% vs. 67.3 ± 41.1%, *P* = 0.073). There was a significant positive relationship between EC and Gal‐3 concentrations (*r*_rm_ = 0.68, *P* = 0.001, *n* = 18; Figure 2d). Distinguishing between exercise modalities, this significant association could be reproduced only in cyclists (*r*_rm_ = 0.79, *P* = 0.006, *n* = 9) and not in runners (*r*_rm_ = 0.61, *P* = 0.063, *n* = 9, data not shown). The association between EC and sST2 concentrations was also significant (*r*_rm_ = 0.57, *P* = 0.010, *n* = 18), but not specific for cyclists (*r*_rm_ = 0.63, *P* = 0.052, *n* = 9) or runners (*r*_rm_ = 0.56, *P* = 0.090, *n* = 9).

### 3.5 Systemic resistances and CTi

Baseline EDFR and SVRi were, on average, higher in cyclists than runners (Table 1), but results were not significantly different. Baseline EDFR and EC proportion and concentration were positively associated (*r* = 0.65, *P* = 0.005; and *r* = 0.75, *P* < 0.001, respectively, both *n* = 17), but these associations could be reproduced only in cyclists (*r* = 0.88, *P* = 0.004; and *r* = 0.87, *P* = 0.006, respectively, *n* = 8) and not in runners (*r* = 0.15, *P* = 0.696; and *r* = 0.39, *P* = 0.301, respectively, *n* = 10). No significant association was found between basal SVRi and circulating cardiac biomarker or EC concentration.

Basal CTi was significantly lower in cyclists compared with runners (*P* = 0.002; Table 1). Acute exercise significantly increased CTi for both exercise modalities (cycling: 70.9 ± 48.4%; running: 61.3 ± 62.3%), with no interaction between groups. A repeated‐measures correlation between CTi and Gal‐3 pre‐exercise –postexercise was also significant (*r*_rm_ = 0.57, *P* = 0.011, *n* = 18), as was the relationship with ECs (*r*_rm_ = 0.51, *P* = 0.038, *n* = 16). These results could not be attributed to one of the exercise modalities, but were significant only when pooling runners and cyclists.

## 4 DISCUSSION

In this study, we tested whether acute high‐intensity interval running and cycling would result in elevated circulating concentrations of novel cardiovascular biomarkers of fibrosis, namely Gal‐3 and sST2, and whether the results would differ between exercise modalities. Furthermore, we investigated the association between said biomarkers and baseline systemic vascular resistances (EDFR and SVRi), exercise‐induced cardiac CTi and sustained endothelial activation (EC shedding). Our results demonstrated that a HIIT session of both exercise modalities induced increases of Gal‐3 and sST2. The concentrations of both biomarkers were significantly related to sustained endothelial activation assessed as the number of circulating ECs. As a possible mechanism, elevated exercise‐induced CTi triggering Gal‐3 secretion from cardiac macrophages (Frangogiannis, 2021), which in turn enhances EC shedding, can be hypothesized.

Although mid‐distance running and cycling are seen as endurance activities regarding the relative isometric and isotonic components of exercise and resulting cardiovascular adaptations (Pelliccia et al., 2018), there is evidence that there might be slight differences between modalities regarding the development of myocardial fibrosis. Tahir et al. (2018) found that myocardial fibrosis determined by cardiac MRI in asymptomatic triathletes was associated with both exercise‐induced hypertension and cycling, but not running distance. Furthermore, left ventricular wall thickness determined by cardiac echocardiography was disproportionately increased in experienced cyclists compared with runners (Fagard et al., 1984). In our study, we found that basal CTi was significantly lower in cyclists. The implication of this finding still has to be elucidated, especially given that Maceira et al. (2023) recently did not find a higher prevalence of coronary calcifications in former professional cyclists compared with matched control subjects. In animal models of heart failure, however, a decrease of myocardial contractility has been associated with health deterioration and the onset of fibrosis (de Souza Vilarinho et al., 2010).

In comparison to running, data of acute exercise‐induced Gal‐3 and sST2 in cycling athletes are sparse (Le Goff, Farre Segura, et al., 2020). Our results extend the present knowledge by showing that acute high‐intensity cycling and running both increased Gal‐3 and sST2 levels postexercise (40% and 8%, respectively), but within normal reference limits (Agnello et al., 2017; Biaggi et al., 2019). Although the exercise‐induced differences are not enormous, they are, nonetheless, interesting when compared with median reductions in Gal‐3 and sST2 initiated by cardiac rehabilitation (−6%, *P* < 0.0001, *n* = 107; and −7%, *P* = 0.035, *n* = 97, respectively), which were considered clinically meaningful (Billebeau et al., 2017).

Recent findings have indicated that the endothelium plays a crucial role in the inflammatory response and pathogenesis of fibrosis. In the early phase, activated endothelium can recruit and stimulate leucocytes, thus perpetuating tissue inflammation, and sustained endothelial activation might contribute to endothelial‐to‐mesenchymal transition in organs such as the lung (Jia et al., 2021) or heart. Acute exercise is a short‐term stressor for the endothelium, triggering EC detachment and an increase of ECs in the peripheral blood (Kropfl et al., 2021). High shear stress, typically occurring in straight segments of vessels, protects against pathological stimuli. In contrast, low oscillatory flow, with changes in direction and magnitude, induces a pro‐inflammatory, profibrotic state (Kruger‐Genge et al., 2019). Runners show lower blood viscosity, shear rate and pressure upon the endothelial walls at higher exercise intensities (Johnson et al., 2012). This could diminish any retrograde (oscillatory) shear stress induced, which is why we would have expected sustained endothelial activation to be more prominent in cyclists. However, our data supported this hypothesis only by showing a statistical trend.

Circulating Gal‐3 is responsible for myocardial vascular remodelling by supporting the formation of collagen by fibroblasts (Hrynchyshyn et al., 2013). In this context, the positive association between acute exercise‐induced Gal‐3 concentrations and sustained endothelial activation by the number of circulating ECs is noteworthy, given that the extracellular matrix morphogenesis could be driven through the exposure of activated and proliferating ECs to collagen type I (Davis & Senger, 2005). This putative mechanism could play a role in the onset of different cardiovascular diseases, such as arrhythmogenic cardiomyopathy or acute coronary syndromes (Sygitowicz et al., 2021), sometimes associated with long‐term high‐intensity exercise training (Eberly et al., 2021; Maisch, 2015), and prescreening in athletes at risk has been recommended (Maisch, 2015).

Potential limitations of this study are the relatively small sample size and the inclusion of only male participants (Haid et al., 2022). Owing to the post hoc nature of the investigation and the lack of pressure measurement during the acute exercise bout, data on exercise‐induced EDFR and SVRi are not available. Also, results have to be interpreted with caution, because it is risky to extend conclusions based solely on a brief episode of acute exercise to long‐term pathological endothelial adaptation induced by exercise training.

To conclude, both baseline and acute exercise‐induced circulating Gal‐3 and sST2 were comparable between experienced runners and cyclists. Our results suggest a strong effect of a HIIT session on these new cardiovascular biomarkers in both exercise modalities. However, only Gal‐3 released from cardiac macrophages was probably driven by an enhanced CTi. Both biomarkers were associated with a higher sustained endothelial activation and therefore possible endothelial‐to‐mesenchymal transition. This putative mechanism adds important knowledge about the exercise‐induced development of cardiovascular and, potentially, further fibrotic conditions to the literature, also relative to the exercise modality.

## AUTHOR CONTRIBUTIONS

The experiments were performed partly at the Exercise Physiology Lab (ETH Zurich, Switzerland) and the Division of Hematology (University Hospital Zurich, Switzerland). Julia M. Kröpfl, Fernando G. Beltrami and Christina M. Spengler were responsible for the conception or design of the work. Julia M. Kröpfl, Fernando G. Beltrami, Christina M. Spengler, T. Dieterle, A. Schmidt‐Trucksäss and Hans‐Jürgen Gruber drafted the work or revised it critically for important intellectual content and performed data acquisition and/or analysis. All authors were responsible for interpretation of data for the work. All authors approved the final version of the manuscript and agree to be accountable for all aspects of the work in ensuring that questions related to the accuracy or integrity of any part of the work are appropriately investigated and resolved. All persons designated as authors qualify for authorship, and all those who qualify for authorship are listed.

## CONFLICT OF INTEREST

The authors declare no conflict of interest.

## Data Availability

All data supporting the results presented in the manuscript are included in the manuscript tables and figures.
